# Purification and Characterization of A New Cold-Adapted and Thermo-Tolerant Chitosanase from Marine Bacterium *Pseudoalteromonas* sp. SY39

**DOI:** 10.3390/molecules24010183

**Published:** 2019-01-06

**Authors:** Yu Zhou, Xuehong Chen, Xiao Li, Yantao Han, Yanan Wang, Ruyong Yao, Shangyong Li

**Affiliations:** 1 Department of Pharmacology, College of Basic Medicine, Qingdao University, Qingdao 266071, China; zy18339956716@163.com (Y.Z.); chen-xuehong@163.com (X.C.); lilix0823@163.com (X.L.); sunshine4581@163.com (Y.W.); 2 Central laboratory, Qingdao University, Qingdao 266071, China; yry0303@163.com

**Keywords:** chitosanase, cold-adaptation, thermo-tolerance, chitooligosaccharide

## Abstract

Chitosanases play an important role in chitosan degradation, forming enzymatic degradation products with several biological activities. Although many chitosanases have been discovered and studied, the enzymes with special characteristics are still rather rare. In this study, a new chitosanase, CsnM, with an apparent molecular weight of 28 kDa was purified from the marine bacterium *Pseudoalteromonas* sp. SY39. CsnM is a cold-adapted enzyme, which shows highest activity at 40 °C and exhibits 30.6% and 49.4% of its maximal activity at 10 and 15 °C, respectively. CsnM is also a thermo-tolerant enzyme that recovers 95.2%, 89.1% and 88.1% of its initial activity after boiling for 5, 10 and 20 min, respectively. Additionally, CsnM is an endo-type chitosanase that yields chitodisaccharide as the main product (69.9% of the total product). It’s cold-adaptation, thermo-tolerance and high chitodisaccharide yield make CsnM a superior candidate for biotechnological application to produce chitooligosaccharides.

## 1. Introduction

Chitosan, a natural cationic polysaccharide, is a linear heteropolymer made of β (1–4) linked Nacetyl-d-glucosamine (GlcNAc or A) and D-glucosamine (GlcN or D). It is produced by the deacetylation of chitin which is the second-most ubiquitous polysaccharide in nature [1,2,3]. Chitosan polymers are widely used as absorbable surgical suture, artificial skin and wound healing accelerators mainly due to their high viscosity and nontoxicity [4,5,6].

Chitosanase (EC.3.2.1.132) is a type of glycoside hydrolase (GH) that catalyzes the hydrolysis of β-1,4-linked glycosidic bond, releasing chitooligosaccharides (CHOS) as main products. CHOS have gained considerable attention owing to their role in several important biological processes, such as antibacterial activity [7], anti-inflammatory activity [8,9], antioxidant activity [10,11,12], anti-allergic activity [13], immuno-enhancer effects [14] and antitumor effects [15,16]. Interestingly, the chemical structures and molecular sizes of the hydrolase products have a significant impact on these biological activities. Thus far, several thousands of chitosanases have been found in bacteria, fungi and plants [17]. In the Carbohydrate-Active Enzymes database (CAZy), chitosanases are classified into six glycoside hydrolases (GH) families: 5, 7, 8, 46, 75, and 80 [18]. Although various chitosanases have been characterized, the enzymes with special features are rather rare. Industrial production often requires enzymes with special properties, such as cold-adaptation, thermo-tolerance and single product distribution [19]. Cold-adapted enzymes can run biocatalytic processes at low temperatures, often saving energy and production costs, and also reducing contamination risks. Thermo-tolerance enzymes have several advantages over thermolabile enzymes, such as low production cost and high degradation efficiency. Especially in chitosan degradation, thermo-tolerance chitosanases could persist the degradation of a substrate at high temperature, which could reduce the viscosity of chitosan substrate. Meanwhile, high proportion product in a mixture of products will be propitious to the purification of chitooligosaccharides. Considering the short of mercantile enzymes with superior properties, it is rather important to find a chitosanase with exceptional properties.

Herein, a new chitosanase, CsnM, was purified from the marine bacterium *Pseudoalteromonas* sp. SY39. CsnM is a cold-adapted enzyme, which also shows excellent thermo-tolerance property. It is an endo-type chitosanase that yields chitodisaccharide as the main product, such excellent properties making CsnM a superior candidate in industrial applications for the production of chitooligosaccharides.

## 2. Results and Discussion

### 2.1. Identification of Strain SY39

**Figure 1:**
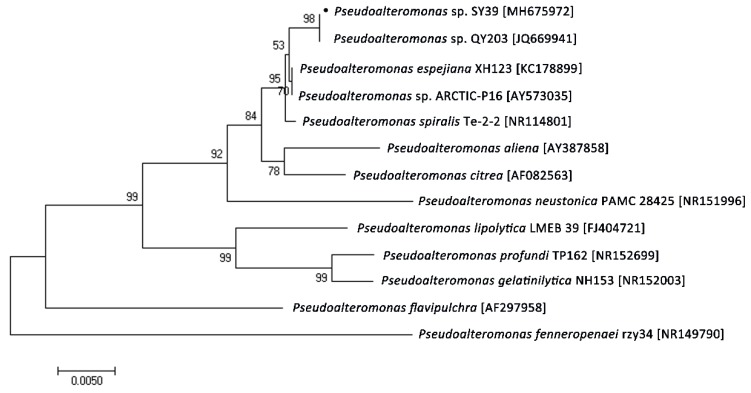
Phylogenetic relationship of the strain SY39. The evolutionary history was inferred using the maximal parsimony analysis of the 16S rDNA sequences. The 16S rDNA sequence was obtained by using the ClustalX and BLASTn programs, respectively. Phylogenetic analysis was carried out using MEGA 7.0 software.

Through sole carbon source screening, five strains with high chitosanase activity (>100 U/mL) were screened and detected. SY39 was selected for further research as it showed the highest activity in these strains. Its highest chitosanase activity (370 U/mL) was determined in the culture supernatant after 72 h of culturing. Molecular identification of strain SY39 was by cloning and sequencing a 1411 bp fragment of its 16S rDNA gene (Genbank accession number: MH675972). The alignment of 16S rDNA gene sequences showed that strain SY39 was 99% identical to the *Pseudoalteromonas* strain L2. According to the phylogenetic position of its 16S rDNA (Figure 1), SY39 was assigned to the genus *Pseudoalteromonas* and named *Pseudoalteromonas* sp. SY39.

### 2.2. Purification and Characterization of CsnM

**Figure 2:**
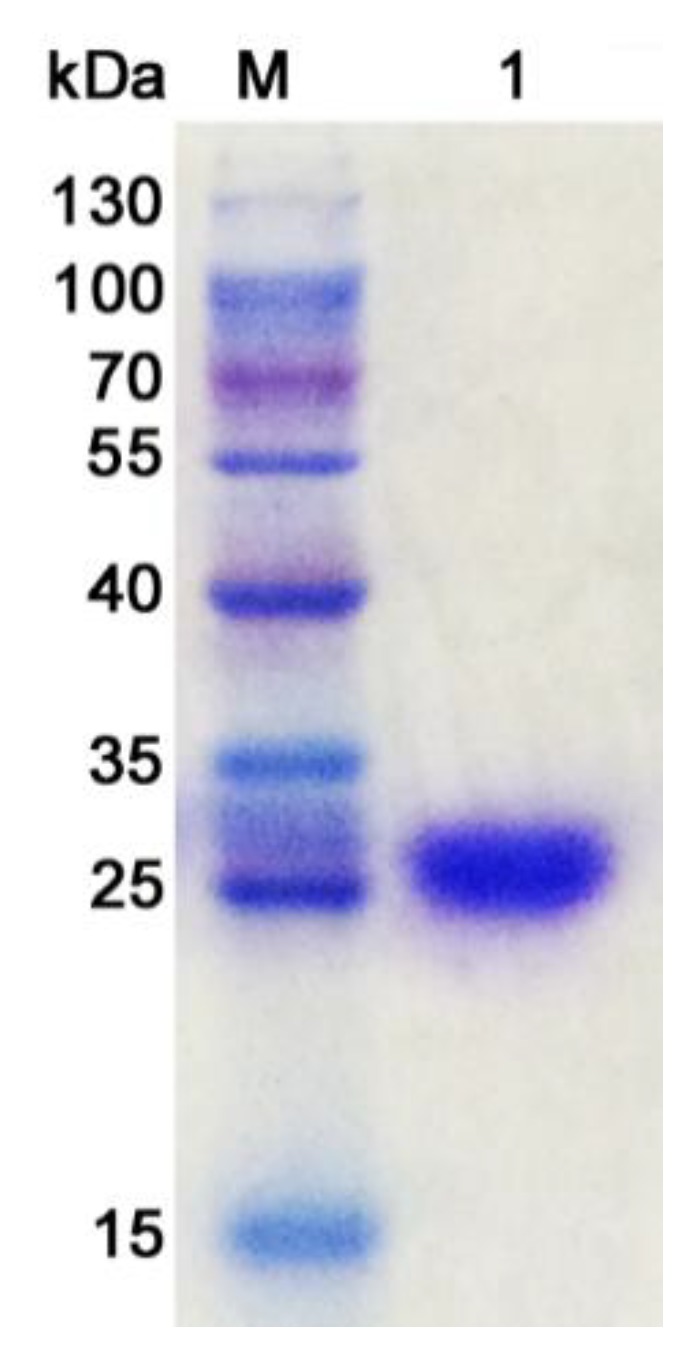
Sodium dodecyl sulfate-polyacrylamide gel electrophoresis (SDS-PAGE) analysis of purified CsnM. (M, molecular marker; 1, purified CsnM).

CsnM was purified 2.4-folds by DEAE-Sepharose column chromatography and its molecular weight was determined to be about 28 kDa by the method of sodium dodecyl sulfate-polyacrylamide gel electrophoresis (SDS-PAGE) (Figure 2). The specific activity of purified CsnM was 393.2 U/mg. The activity recovery was 60.5%, and about 220,000 U (0.56 g) chitosanases could be obtained from 1 L culture supernatant of strain SY39.

**Figure 3:**
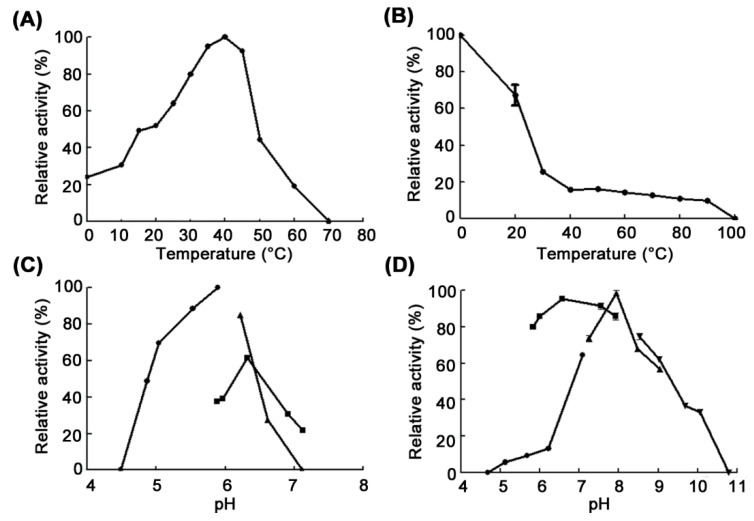
Effect of temperature and pH on the activity of CsnM. (**A**) Effect of temperature on the activity of CsnM. (**B**) Effect of temperature on the thermostability of CsnM. Residual activities were detected at 40 °C. The activity of the pre-incubated CsnM at 4 °C was taken as 100%. (**C**) Effect of pH on the activity of CsnM. The optimal pH was analyzed by detecting activity in sodium acetate buffer (pH 4.49–5.89, solid round), phosphate buffer (pH 5.88–7.12, solid square) and Tris-HCl buffer (pH 6.22–7.11, solid triangle). (**D**) Effect of pH on the stability of CsnM. The pH stability was determined by detecting the residual activity after CsnM was pretreated in different buffers at 4 °C for 24 h, including Na_2_HPO_4_-citric acid buffer (pH 4.69–7.09, solid round), phosphate buffer (pH 5.83–7.92, solid square), Tris-HCl buffer (pH 7.24–9.04, solid triangle) and glycine-NaOH buffer (pH 8.53–10.78, solid inverted triangle). Experiments were conducted three times.

The effect of temperature and pH on chitosanase activity was determined using the purified CsnM (Figure 3). CsnM showed the maximum activity at 40 °C and showed 96% of this activity at 45 °C. Meanwhile, it showed 24.1%, 30.6% and 49.4% of its maximum activity at 0, 10 and 15 °C, respectively (Figure 3A). This result indicates that CsnM is a cold-adapted enzyme. Cold-adapted enzymes can run biocatalytic processes at low temperature and controllably hydrolyze substrate at mild reaction conditions. Although a number of chitosanases have been reported, only two of them have been shown to have cold-adapted properties, chitosanases from *Janthinobacterium* sp. 4239 [20] and *Gynuella sunshinyii* [21]. Cold-adapted chitosanase from *Janthinobacterium* sp. 4239 showed its maximum activity at 45 °C and 30% to 70% of the maximum activity at 10–30 °C. Another cold-adapted chitosanase, GsCsn46A from *Gynuella sunshinyii*, showed its maximum activity at 30 °C and showed 70% of its maximum activity at 15 °C. Compared with their mesophilic homologs, cold-adapted enzymes usually have lower thermostability. In this study, CsnM retained only 25.4% and 15.8% of its initial activity after incubation at 30 and 40 °C for 1 h, respectively (Figure 3B). The optimum pH for CsnM was determined to be pH 5.9 in sodium acetate buffer (Figure 3C). CsnM retained over 80% of its original activity after incubation at pH ranging from 5.83 to 7.92 at 4 °C for 24 h (Figure 3D). 

The effects of metal ions on the activity of CsnM are shown in Table 1. Purified CsnM was slight slightly active in the presence of Mg^2+^, Li^+^, K^+^, Ca^2+^, Ba^2+^ and significantly inhibited in the presence of Al^3+^, Cu^2+^, Ni^2+^, Fe^2+^, Co^2+^, Zn^2+^. The addition of 0.001–0.5M NaCl increased the CsnM activity to 128% to 151%. Chemical reagents such as ethanol, isopropanol and dimethyl sulfoxide had no significant effect on the activity of CsnM.

### 2.3. Thermo-Tolerance Property of CsnM

**Figure 4:**
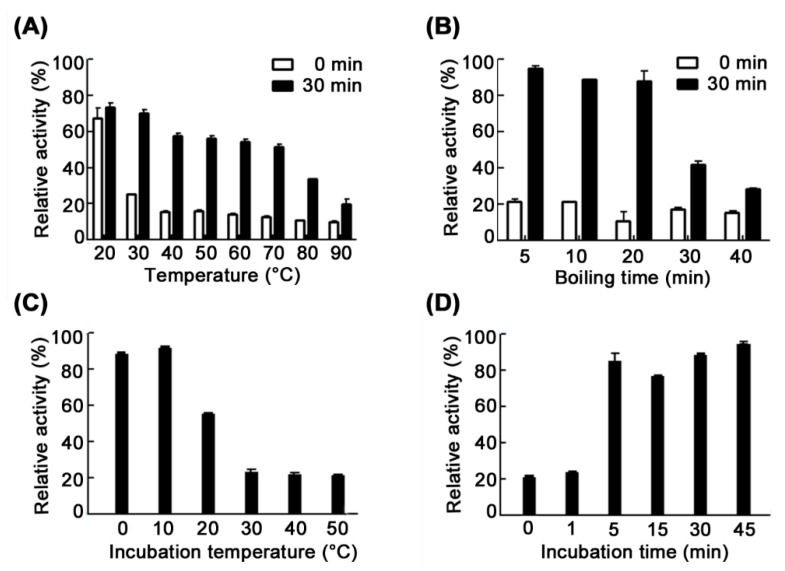
Thermo-tolerance of CsnM. (**A**) The difference in thermostability of CsnM incubated at 0 °C for 0 and 30 min. (**B**) Effect of boiling time on the activity recovery of CsnM after heat treatment. (**C**) Effect of incubation temperature on the activity recovery of CsnM after heat treatment. (**D**) Effect of incubation time on the activity recovery of CsnM after heat treatment. The activity of unheated CsnM was used as control (100%). Values are the means of three independent experiments ± standard deviations.

When we explored the thermostability of CsnM, we found an interesting phenomenon. The thermostability of CsnM showed a great difference between incubation at 0 °C for 0 and 30 min after heat treatment for 1 h (Figure 4A). CsnM retained only 25.4% and 15.8% of its initial activity at 30 and 40 °C respectively, when directly determined after heat treatment for 1 h. However, when CsnM was incubated at 0 °C for 30 min, the residual activity could recover to 70% and 57.8% under the same heat treatment conditions. Moreover, CsnM also recovered 33.7% and 19.8% of its initial activity after incubation at 80 and 90 °C for 1 h, respectively.

To further determine the thermo-tolerant properties of CsnM, the enzyme was boiled for 5, 10, 20, 30 or 40 min and immediately incubated at 0 °C for 30 min. The results indicated that CsnM could recover 95.2%, 89.1% and 88.1% of its initial activity after boiling for 5, 10 and 20 min, respectively. Even when the boiling time was prolonged to 30 and 40 min, CsnM could still recover 42.1% and 28.7% of its initial activity, respectively (Figure 4B). To find the optimal incubation temperature, CsnM was immediately incubated for 30 min at diverse temperatures (0–50 °C) after boiling for 10 min. The activity of CsnM recovered and reached 21.8% to 92.3% after incubation at 0–50 °C for 30 min (Figure 4C). Maximal activity recovery (92.3%) was observed when the boiled CsnM was incubated at 10 °C (Figure 4C). Further, to obtain the optimal incubation time, after boiling for 10 min, CsnM was immediately incubated at 10 °C for various times (1–45 min). These results indicated a gradual recovery of chitosanase activity with extended incubation times (Figure 4D). Extended incubation times (up to 10 h) led to a small reduction in the recovered activity (data not shown). As mentioned earlier, other chitosanases have not been reported to be thermo-tolerant. The properties of thermo-tolerance are beneficial to the storage and transport of enzymes since the heat-killed enzyme could recover some of its activity after incubation at low temperature. As far as we know, CsnM is the first reported chitosanase with thermo-tolerance properties. Thermo-tolerance property always contribute to expanding its application. In addition, enzymes combined with nanomaterials or metal ions using immobilization technology could improve its stability and reuse in an industrial process [22,23,24]. In our further work, we intend to immobilize the enzyme to make it more stable.

### 2.4. Hydrolytic Pattern and Reaction Products of CsnM

**Figure 5:**
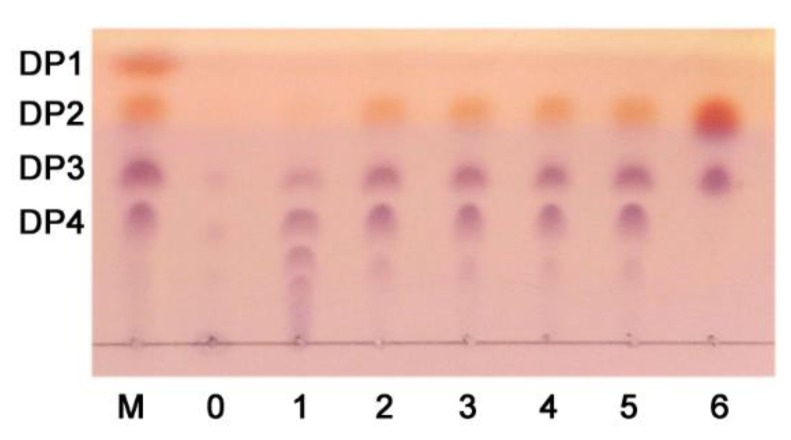
Thin-layer chromatography (TLC) analysis of chitosanase-hydrolytic products. Lane M: standard chitosan oligomers (GlN, DP1–4); Lanes 0–6: enzymatic hydrolysates of chitosan incubated at 30 °C for 0, 1, 10, 30, 120, 360 and 1440 min, respectively.

The hydrolytic pattern of CsnM was observed by thin-layer chromatography (TLC) assay (Figure 5). Monosaccharide (DP1) was not detected in the final products, which indicated that CsnM was not an exo-type enzyme. Moreover, the proportion of chitooligosaccharides with high DP (degree of polymerization) gradually decreased during the incubation period. However, the relative proportions of chitooligosaccharides with low DP, such as DP2 and DP3, gradually increased. The rapid depolymerization, the increase of dispersion and the composition of final products indicated that CsnM acted in an endolytic manner.

**Figure 6:**
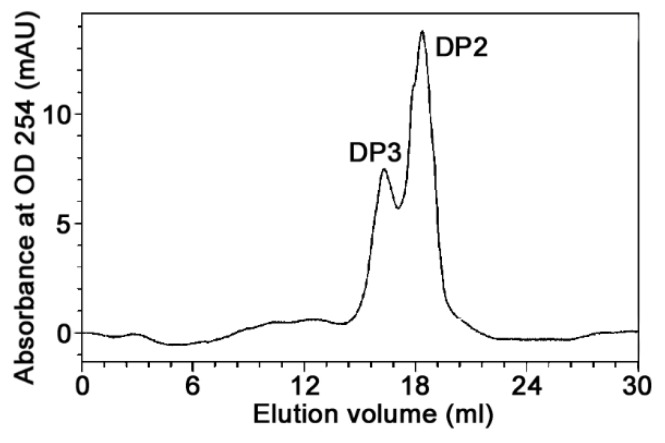
High performance liquid chromatography (HPLC) analysis of the end product of CsnM.

After complete degradation of chitosan by CsnM, only two clear spots on the TLC plate could be observed (Figure 5). The mobility ratio of these spots were in good agreement with the chitosandisaccharide and chitosantrisaccharide markers. The final degradation product was also analyzed by high performance liquid chromatography (HPLC) with a superdex peptide 10/300 column (Figure 6). The result indicated that chitosan-disaccharides were the main product (69.9% of the total product). High proportion product in a mixture of products will be propitious to the purification of chitooligosaccharide. Thus far, the main products of most of the reported chitosanases such as enzymes from *Aspergillus* sp. W-2 [25], *Bacillus cereus* GU-2 [26], *Bacillus cereus* D-11 [27], have been a mixture of DP2–DP5. These mixtures were quite hard to separate. The main products of hydrolysis released from *Purpureocillium lilacinum* CFRNT12 were chitosan-tridisaccharide (DP3) and chitosan-tetradisaccharide (DP4) after 24 h (43.91% and 50.36%) of reaction [28]. Although the end product of chitosanase from Paenibacillus dendritiformis was only chitosan-tridisaccharide (DP2), however, it showed a low specific activity (76.4 U/mg) [29].

## 3. Materials and Methods

### 3.1. Isolation and Identification of Strains SY39

The marine bacterium *Pseudoalteromonas* sp. SY39 was isolated from the Yellow Sea sediment (Qingdao, China), grown in chitosan selective medium [30] and the activity of chitosanase was detected. The 16S rDNA gene of strain SY39 was amplified using 27F and 1492R primer in accordance with the method described by Li et al. [31]. 16S rDNA gene sequence of strain SY39 was used to obtain the closely related sequence from GenBank using the BLASTn program (National Center of Biotechnology Information, Bethesda, MD, USA). Multiple sequence alignment was performed using the CLUSTAL X program (Conway Institute UCD Dublin, Dublin, Ireland). The phylogenetic trees of these 16S sequence were constructed using MEGA 7.0 (https://www.megasoftware.net/download_form).

### 3.2. Purification of CsnM

Strain SY39 was cultured in 100 mL chitosan selective medium at 25 °C in a shaker (180 rpm) for 72 h. After centrifugation at 12,000× *g* for 10 min, the culture supernatant of strain SY39 was obtained and loaded onto a DEAE Sepharose 6B column (1.6 × 5 cm) at AKTA avant 150 platform. Elution was carried out with a linear gradient of NaCl (0–1 M) at a flow rate of 3 mL/min. When the concentration of NaCl reached to 0.3 M, the activity of CsnM was determined and obtained for further research. SDS-PAGE was used to analyze the molecular weight of CsnM [32]. The coomassie brilliant blue R250 was used to stain the protein band. The concentration of purified CsnM was determined by BCA method using bovine serum albumin (BSA) as standard.

### 3.3. Effect of Temperature, pH, Metal Ions and Chelators on CsnM

The optimal temperature of CsnM was determined by assaying its activity at 0–70 °C. To determine its thermo-stability, CsnM was incubated at a wide range of temperatures from 0 to 100 °C for 1 h. Afterwards, residual activity was measured at 40 °C. The optimal pH of CsnM was measured using different buffers in the reaction system, including sodium acetate buffer (50 mM, pH 4.49–5.9), phosphate buffer (50 mM, pH 5.88–7.12) and Tris-HCl buffer (50 mM, pH 6.22–7.11). To determine its pH stability, the residual activity was measured after CsnM was incubated in the pH range of 4.69–10.78 with the different buffers for 24 h at 4 °C; the buffers used were, Na_2_HPO_4_-citric acid buffer (50 mM, pH 4.69–7.09), phosphate buffer (50 mM, pH 5.83–7.92), Tris-HCl buffer (50 mM, pH 7.24–9.04) and glycine-NaOH buffer (50 mM, pH 8.53–10.78). The effect of metal ions and chelators on CsnM was inspected by monitoring the enzymatic activity in the presence of various metal ions or chelators (1 mM).

### 3.4. Thermo-Tolerance Properties of CsnM

To determine the thermo-tolerance properties of CsnM, its residual activity was determined after heat treatment, followed immediately by incubation at a low temperature. Purified CsnM was first boiled for 5, 10, 20, 30 or 40 min. Following boiling, the enzyme was immediately incubated for 1, 5, 15, 30 or 45 min at 0–50 °C. After that, residual activities were determined at the optimal conditions. As a control, after boiling, residual activity was immediately determined to confirm the influence of low temperature incubation. The activity of chitosanase without heat treatment was taken as 100%. 

### 3.5. Measurement of CsnM Activity

The activity of chitosanase was determined by 3,5-dinitrosalicylic acid (DNS) method [33]. The reaction was carried out with 50 μL properly diluted enzyme solution and 450 μL substrate solution (0.3% (*w*/*v*) soluble chitosan, pH 5.9) at 40 °C for 10 min. Following this, 375 μL of DNS reagent was added. The mixture was boiled for 10 min, cooled to room temperature and centrifuged to remove the precipitate. One unit of chitosanase activity was taken as the amount of enzyme that released 1 μmol reduced sugar per min. d-glucosamine was used for generating the standard curve.

### 3.6. Action Pattern and End Product

To determine the action pattern of CsnM, chitosan (3 mg/mL) by CsnM (300 U/mL) was analyzed using TLC at various reaction times (0, 1, 10, 30, 120, 360, 1440 min). Reaction products on a HPTLC plate developed with n-butanol/water/glacial acetic acid (2:1:1, by vol). The sugars on the plates were visualized by spraying with 0.5% ninhydrin dissolved in ethanol and heating to 80 °C for 20 min. To obtain the end products, the enzyme (300 U) was mixed into 10 mL of substrate solution (3 mg/mL) and cultured at 30 °C for 24 h. To examine the components of the end product, a HPLC with a Superdex peptide 10/300 gel filtration column (GE Healthcare, Madison, WI, USA) was applied. Then, 100 µL chitooligosaccharide was added with a volume (5 µL) of 0.1 M 2-AMAC solution (dissolved in a 17:3 (*v*/*v*) mixture of dimethyl sulfoxide and glacial acetic acid) and 1 M sodium cyanoborohydride (100 µL). The mixture was incubated at 90 °C for 40 min in the dark and the reaction was stopped at −20 °C [34]. Finally, 500 µL of 50% dimethyl sulfoxide was added and centrifuged for HPLC. The mobile phase (flow rate, 0.6 mL/min) was 0.2 M ammonium bicarbonate. The detection wavelength was 254 nm.

## 4. Conclusions

In this study, CsnM, a chitosanase from the marine bacterium *Pseudoalteromonas* sp. SY39, was purified and characterized. Interestingly, CsnM is a thermo-tolerant enzyme that could recover 95.2%, 89.1% and 88.1% of its initial activity after boiling for 5, 10 and 20 min, respectively. Meanwhile, it is also a cold-adapted enzyme that produces chitodisaccharides as the main products. These excellent properties make CsnM a superior candidate for biotechnological application. Future work will focus on its gene cloning, relationship of structure and function, and immobilization technology.

## Figures and Tables

**Table 1:** Effects of metal ions and chelators on the activity of CsnM.

Reagent Added	Concentration (mM)	Relative Activity (%)
None	-	100 ± 0.1
NaCl	1	128 ± 1.3
	5	130 ± 4.6
	25	138 ± 1.8
	50	143 ± 1.7
	100	151 ± 2.0
	250	137 ± 1.6
	500	135 ± 1.1
Li_2_SO_4_	1	119 ± 0.7
KCl	1	115 ± 0.7
MgSO_4_	1	113 ± 1.5
BaCl_2_	1	105 ± 0.3
CaCl_2_	1	117 ± 1.3
NiCl_2_	1	74 ± 2.0
CoCl_2_	1	72 ± 0.3
FeSO_4_	1	70 ± 1.7
ZnCl_2_	1	36 ± 1.5
CuSO_4_	1	14 ± 0.1
FeCl_3_	1	10 ± 0.7
AlCl_3_	1	9 ± 0.8
EDTA	1	65 ± 2.8
SDS	1	54 ± 8.7
Ethanol	5	98 ± 1.4
	50	105 ± 2.2
	100	98 ± 4.2
Isopropyl alcohol	5	98 ± 1.9
	50	102 ± 2.3
	100	102 ± 1.7
DMSO	5	100 ± 0.2
	50	98 ± 0.2
	100	100 ± 3.1
